# Quantitative analysis of treatments using real‐time image gated spot‐scanning with synchrotron‐based proton beam therapy system log data

**DOI:** 10.1002/acm2.13029

**Published:** 2020-11-05

**Authors:** Takaaki Yoshimura, Shinichi Shimizu, Takayuki Hashimoto, Kentaro Nishioka, Norio Katoh, Hiroshi Taguchi, Koichi Yasuda, Taeko Matsuura, Seishin Takao, Masaya Tamura, Sodai Tanaka, Yoichi M. Ito, Yuto Matsuo, Hiroshi Tamura, Kenji Horita, Kikuo Umegaki, Hiroki Shirato

**Affiliations:** ^1^ Department of Health Sciences and Technology Faculty of Health Sciences Hokkaido University Sapporo Japan; ^2^ Proton Beam Therapy Center Hokkaido University Hospital Sapporo Japan; ^3^ Department of Radiation Medical Science and Engineering Faculty of Medicine Hokkaido University Sapporo Japan; ^4^ Global Station for Quantum Medical Science and Engineering Global Institution for Collaborative Research and Education (GI‐CoRE) Hokkaido University Sapporo Japan; ^5^ Department of Radiation Oncology Faculty of Medicine Hokkaido University Sapporo Japan; ^6^ Faculty of Engineering Hokkaido University Sapporo Japan; ^7^ Department of Statistical Data Science The Institute of Statistical Mathematics Tokyo Japan; ^8^ Department of Proton Beam Therapy Research Center for Cooperative Projects Faculty of Medicine Hokkaido University Sapporo Japan

**Keywords:** spot‐scanning proton beam therapy, organ motion, beam delivery efficiency, treatment time

## Abstract

A synchrotron‐based real‐time image gated spot‐scanning proton beam therapy (RGPT) system with inserted fiducial markers can irradiate a moving tumor with high accuracy. As gated treatments increase the beam delivery time, this study aimed to investigate the frequency of intra‐field adjustments corresponding to the baseline shift or drift and the beam delivery efficiency of a synchrotron‐based RGPT system. Data from 118 patients corresponding to 127 treatment plans and 2810 sessions between October 2016 and March 2019 were collected. We quantitatively analyzed the proton beam delivery time, the difference between the ideal beam delivery time based on a simulated synchrotron magnetic excitation pattern and the actual treatment beam delivery time, frequency corresponding to the baseline shift or drift, and the gating efficiency of the synchrotron‐based RGPT system according to the proton beam delivery machine log data. The mean actual beam delivery time was 7.1 min, and the simulated beam delivery time in an ideal environment with the same treatment plan was 2.9 min. The average difference between the actual and simulated beam delivery time per session was 4.3 min. The average frequency of intra‐field adjustments corresponding to baseline shift or drift and beam delivery efficiency were 21.7% and 61.8%, respectively. Based on our clinical experience with a synchrotron‐based RGPT system, we determined the frequency corresponding to baseline shift or drift and the beam delivery efficiency using the beam delivery machine log data. To maintain treatment accuracy within ± 2.0 mm, intra‐field adjustments corresponding to baseline shift or drift were required in approximately 20% of cases. Further improvements in beam delivery efficiency may be realized by shortening the beam delivery time.

## 1 INTRODUCTION

In proton beam therapy, it is known that interplay effects may arise, and the baseline of an internal tumor location may change during beam delivery.^1^ Compared to the conventional passive scattering method, scanning methods have greater sensitivity to interplay effects such as baseline shift or drift due to breathing, heartbeats, or intestinal activity, which can lead to an inhomogeneous target dose distribution (e.g., hot or cold spots) in the absence of motion mitigation techniques.^2^, ^3^, ^4^, ^5^, ^6^, ^7^ Thus, interplay effects must be considered when treating a moving tumor with scanning proton beam delivery.

Various procedures have been developed to mitigate the effects of interplay on target dose distribution. Clinical approaches include respiratory gating using surrogate markers^2^, ^6^, ^8^ and tumor tracking using implanted fiducial markers.^7^, ^9^, ^10^, ^11^, ^12^ Respiratory gating with surface markers uses the relationship between internal motion and surface markers or respiratory holding and surface motion.^2^, ^6^, ^8^ Although monitoring the body surface is a valid surrogate for target motion in tumors close to the surface, such as those in the breast, these methods are vulnerable to intra‐fractional changes in the relationship between the internal tumor motion and external surface.^13^, ^14^, ^15^ Tumor tracking with implanted fiducial markers uses the relationship between the internal marker coordinates and planned marker coordinates calculated from treatment planning with computed tomography (CT).^7^, ^9^, ^10^, ^11^, ^12^

Since 2014, we have been clinically operating a synchrotron‐based real‐time image gated spot‐scanning proton beam therapy (RGPT) system,^7^, ^10^, ^11^ which is based on the X‐ray real‐time tumor‐tracking radiation therapy (RTRT) system developed by Shirato et al. in 1999.^16^ The synchrotron‐based RGPT system uses two orthogonal sets of X‐ray fluoroscopes, and the target is irradiated only when the measured marker position is within ±2.0 mm of the planned marker position.^12^, ^17^ The synchrotron‐based RGPT system can reduce the irradiation volume by 50%–75%, which represents a significant reduction in the irradiation of normal tissue around the target.^7^

Although the synchrotron‐based RGPT system is effective for motion management during radiotherapy, there are concerns over the fact that the beam delivery time is prolonged. Since it is important for many scanning proton beam delivery facilities to consider the treatment room throughput and efficiency, Suzuki et al. evaluated the treatment process time with passive scattering and spot‐scanning proton beam delivery.^18^, ^19^ Yoshimura et al. analyzed the treatment process time during radiation therapy using synchrotron‐based RGPT system log data.^20^ In many proton beam therapy facilities, the daily treatment schedule for patients is divided into fixed 30‐min time slots.^18^, ^19^

It is important to clarify the efficiency of beam delivery for the irradiation of moving targets. Tsunashima et al. demonstrated the efficiency of synchrotron‐based respiratory‐gated proton irradiation to treat moving targets.^6^ They compared the beam delivery times required to deliver 100 MU for nongated irradiation, respiratory‐gated irradiation at a 30% duty cycle around peak exhalation with a fixed magnetic excitation cycle, and respiratory‐gated irradiation at a patient’s respiratory cycle with a variable excitation cycle. Depending on the selected proton beam delivery parameters, the average beam delivery time for respiratory‐gated irradiation was two to five times longer than nongated proton beam delivery. However, to the best of our knowledge, the gating efficiency of synchrotron‐based implanted marker gated proton beam delivery has not been analyzed.

Thus, motion management is important for proton therapy of a moving tumor. The synchrotron‐based RGPT system can irradiate a moving tumor with high accuracy. Gated treatment can have a large effect on treatment time, and this could conflict with the 30 min scheduled per treatment session. The aim of this study was to determine the frequency of intra‐field adjustments corresponding to baseline shift or drift and the proton beam delivery efficiency based on our clinical experience. The results should be beneficial for many proton therapy facilities planning to introduce implanted marker gated proton beam therapy.

## 2 MATERIALS AND METHODS

### 2.1 Patient data

The quantitative analysis in this study included 118 patients who had previously received spot‐scanning proton therapy at our institution between October 2016 and March 2019. We categorized patients based on the disease site: liver, pancreas, lung, prostate, and adrenal gland. In this study, we focused on gated irradiation. The number of patients was 67 for prostate, 39 for liver, seven for pancreas, four for lung, and one for the adrenal gland. It accounts for 53.2% of all patients treated with proton beam therapy at our facility during the study period. This study was approved by the ethics committee of our hospital (016‐0454).

### 2.2 Treatment planning

All patients received a planning CT scan with a slice thickness of 1.25 or 2.5 mm performed on a 16‐slice CT scanner (Optima CT580 W, GE Healthcare, Waukesha, WI). All structures were contoured using the Pinnacle3 treatment planning system (TPS; ver.9.0, Philips, Inc., Madison, WI). Assuming tumor‐tracking proton beam treatment with PROBEAT‐RT (Hitachi, Ltd., Tokyo, Japan), treatment planning for all the patients was performed using VQA TPS (Hitachi, Ltd., Tokyo, Japan) with two optimization methods. The first was single field uniform dose optimization (SFUD), and the other was multifield optimization, called intensity modulated proton therapy (IMPT), with robust optimization. In the dose calculation, SFUD was generally used. IMPT with robust optimization was used for cases where it was difficult to satisfy the dose constraints. All structures were transferred from Pinnacle^3^ to VQA via Digital Imaging and Communications in Medicine (DICOM).^21^

Similar to photon therapy, previous studies have reported that when a simple geometric expansion of the clinical target volume (CTV) is used in proton therapy, there is insufficient dose coverage of the target because of proton range uncertainty.^22^, ^23^, ^24^ Thus, beam‐specific optimization margins for the CTV have been recommended for proton treatment planning. In this study, all treatment plans used the beam‐specific margin derived from the range uncertainty calculated as 3.5% of the depth of the distal and proximal edges in water equivalent length.^21^, ^25^

To evaluate the planning robustness related to patient positioning, we simulated the dose distribution by shifting the isocenter for six axes (left‐right (L‐R), anterior‐posterior (A‐P), and superior‐inferior (S‐I)) on the planning CT.^26^, ^27^ At our institution, the shift tolerance for each direction was set to a maximum of 8 mm for the liver and a maximum of 5 mm for other sites.^20^ During the actual treatment, physicians determined the final patient‐specific tolerance range based on the robust evaluation report and actual tumor motion observed in the fluoroscopy image.

**Fig. 1:**
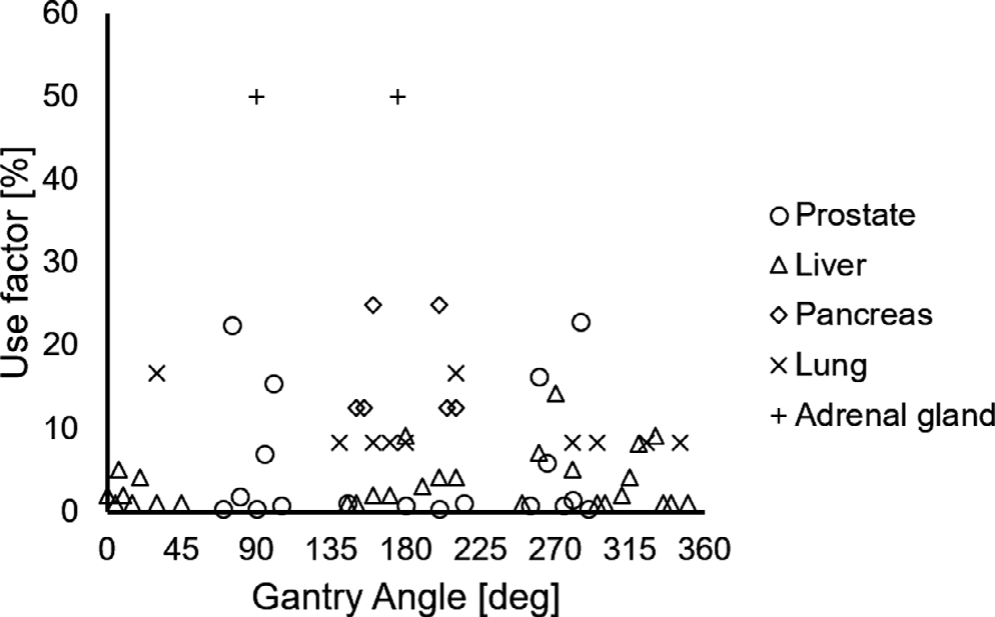
Use factor of gantry angles for each category.

Table 1 lists the characteristics of the treatment plans for each category in this study, including the prescribed dose, fraction, number of plans, number of plans using a short‐range applicator, number of sessions, number of fields per session, optimization method, and the CTV. We evaluated the use factor of the beam angle as the ratio of the usage number of the beam angle to the total number of fields.^18^ Figure 1 shows the use factors of the gantry angles used in this study.

**Table 1:** Characteristics of the treatment plans in this study

Categories	Prescribed Dose [GyE]	Fraction	Number of plans		Number of sessions	Number of fields per session		Optimization methods		CTV [ml]	
				(SRA)		Mean	Range	SFUD	IMPT	Mean	Range
Prostate	70	30	66		1821	4	2‐4	50	19	68	31.4‐154.5
	63	21	3		47						
Liver	60	10	1		10	2	2‐4	44	1	259.5	1.6‐2246.2
	66	10	7	(3)	70						
	72.6	22	22	(6)	447						
	74	37	5	(3)	20						
	76	20	10	(3)	180						
Pancreas	50	25	1		25	2	2‐2	8	0	213.2	38.6‐382.3
	55	25	7		140						
Lung	70	10	4	(1)	40	3	3‐3	4	0	39.5	4.9‐77.6
Adrenal gland	60	10	1		10	2	2‐2	1	0	28.1	28.1‐28.1
Total			127	(15)	2810			107	20		

SFUD, single field uniform dose; IMPT, intensity modulated proton therapy with robust optimization; CTV, clinical target volume; SRA, short‐range applicator.

### 2.3 Synchrotron‐based real‐time image gated spot‐scanning proton beam therapy (RGPT) system

We used the synchrotron‐based spot‐scanning proton beam system PROBEAT‐RT. The synchrotron beam has a maximum range of 30 g/cm^2^ and an irradiation field size of 30 × 40 cm. In the synchrotron‐based RGPT system, fluoroscopy images obtained from two orthogonal sets of the X‐ray tube and flat panel detectors placed at ±45° relative to the proton beam direction are used to observe static and dynamic tumor locations. Details of the proposed synchrotron‐based RGPT system are provided in a previous report.^10^, ^20^

**Fig. 2:**
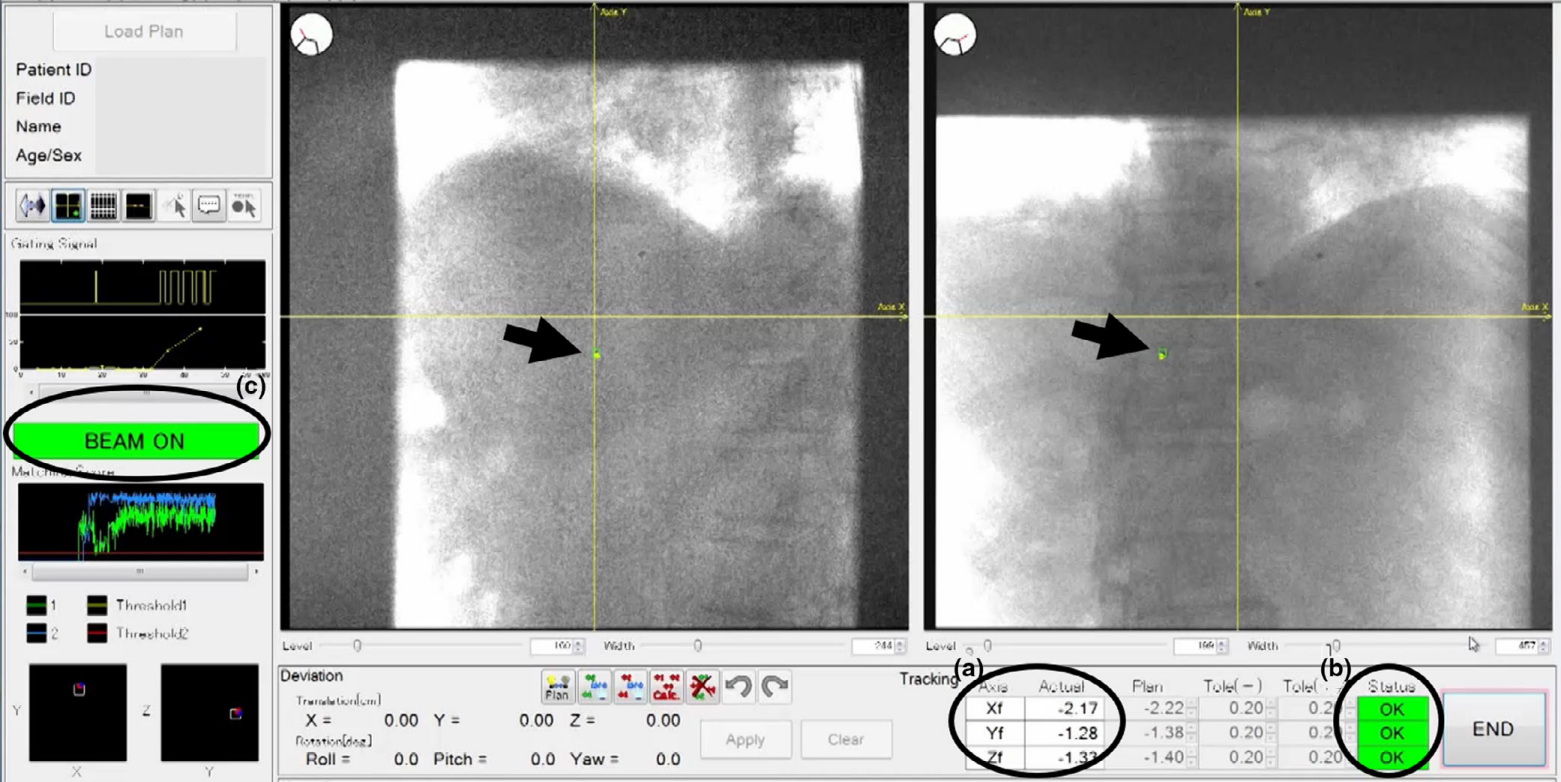
Captured fluoroscopic image during irradiation. This is a real‐time fluoroscopic image that was taken during the treatment of a liver patient with gated irradiation. The arrows show the inserted fiducial markers. (a) Actual three‐dimensional marker position calculated in real time, (b) status when the marker is within the gating window of the planned position for each axis, and (c) gate on/off status.

We used the gating function to manage the internal motion. The synchrotron‐based RGPT system was used if the tumor was within a movable organ such as the lung, liver, pancreas, or prostate. We used a 1.5‐ or 2.0‐mm diameter gold internal fiducial marker near the tumor before the CT scan for treatment planning. The breath hold treatment planning CT was used for dose calculation. For the motion evaluation, we used the 4‐dimensional CT (4DCT) images at treatment planning and the fluoroscopic images during radiotherapy. We checked the location of the marker with two orthogonal sets of X‐ray fluoroscopes during radiotherapy by using real‐time pattern recognition technology for automatic recognition of the projected figure of the gold marker in fluoroscopic images (Fig. 2). The pulse rate for fluoroscopy was 30 or 15 Hz for the liver, pancreas, and lung patients, and 1 Hz for prostate patients.^20^ The ranges of the X‐ray tube voltage and current imaging parameters were 30–125 kV and 20–125 mA, respectively. The imaging parameters depend not only on the patient characteristics of the disease site but also on the beam angle. We set the imaging parameters for each patient and gantry angle as low as possible to reduce the exposure dose from the fluoroscopy imaging.

**Fig. 3:**
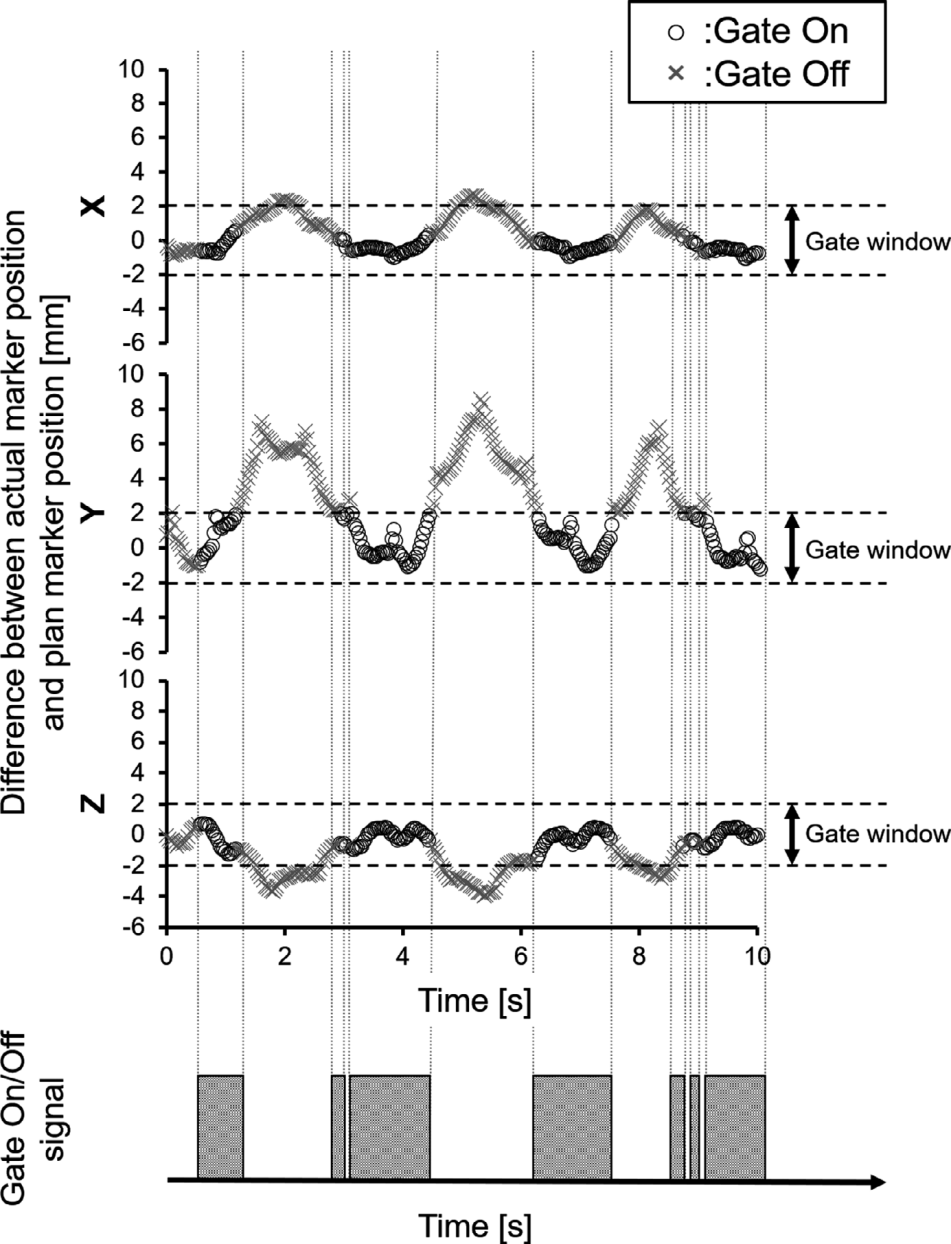
Example gate signals for a liver patient. The gate signal is only turned on when the difference between the actual and planned marker positions is in the gate window (±2.0 mm).

In the synchrotron‐based RGPT system, the proton beam is gated when the marker enters a preassigned gating window. The gating window tolerance for the actual treatment of each patient was set to ±2.0 mm based on our previous study.^7^, ^9^, ^17^, ^28^ Figure 3 shows an example of how the gate signal was recorded for liver patients.

### 2.4 Synchrotron magnetic excitation cycles

**Fig. 4:**
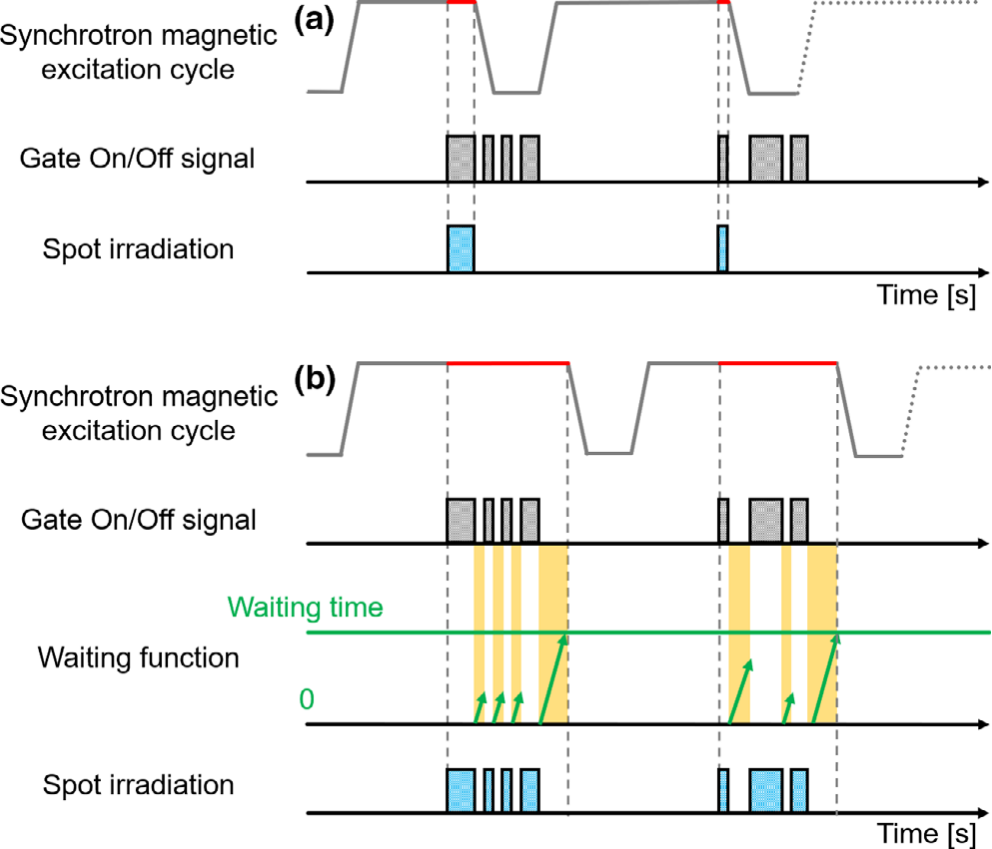
Schematic illustration that shows the difference between the synchrotron magnetic excitation cycle of (a) the previous operation with respiration and (b) operation with multiple gating beam delivery. In the flat top phase of the synchrotron magnetic excitation cycle, the red line shows the period between the start of spot irradiation based on the gate on/off signal and the deceleration phase. In the waiting function, the green arrow indicates the elapsed time between the gate signal being turned off and the next gate signal being turned on.

Fundamentally, the synchrotron magnetic excitation cycles comprise injection, acceleration, flat top, and deceleration phases, as shown in Fig. 4. For synchrotron operation with tumor tracking, a waiting function was installed as a delay gate to improve the irradiation efficiency. If the wait time reached the predefined limit time, the synchrotron magnetic excitation cycle transited from the flat top phase to the deceleration phase. As shown in Fig. 4, spot irradiation can be promptly restarted without deceleration when the elapsed wait time is shorter than the predefined limit time. Thus, compared with the previous synchrotron operation with respiration, this function enables multiple gating beam delivery with a single synchrotron magnetic operation cycle. Commercially, multiple gating beam delivery is used to shorten the beam delivery time for the synchrotron‐based RGPT system as much as possible. We installed this function as a part of the proton beam control to improve the irradiation efficiency of the gating technique. The details on multiple gating beam delivery for synchrotron operation are presented in the previous papers.^7^, ^29^ When the synchrotron is operating, multiple gating beam delivery improves the gate irradiation efficiency and reduces the proton beam delivery time.

### 2.5 Evaluation of the beam delivery time

Beam delivery time can be expressed as a function of the number of fields per session and the target volume in the treatment planning. Suzuki et al. defined the beam delivery time per session for passive scattering or spot‐scanning proton therapy as $T_{\mathrm{BS}}\left(X,V\right)$,^18^, ^19^ where *X* is the number of fields per session and *V* is the CTV in cubic centimeters. Yoshimura et al. redefined the beam delivery time for the RGPT system as $T_{\mathrm{BSR}}\left(X,V,R\right)$ based on the previous definition by Suzuki et al,^18^, ^19^ where *R* is the usage of the gating function.^20^ The transmission and reception of the signal between the beam delivery machine and the control device are recorded chronologically in treatment log data. The details of the treatment process calculation are described in our previous report.^20^

**Fig. 5:**
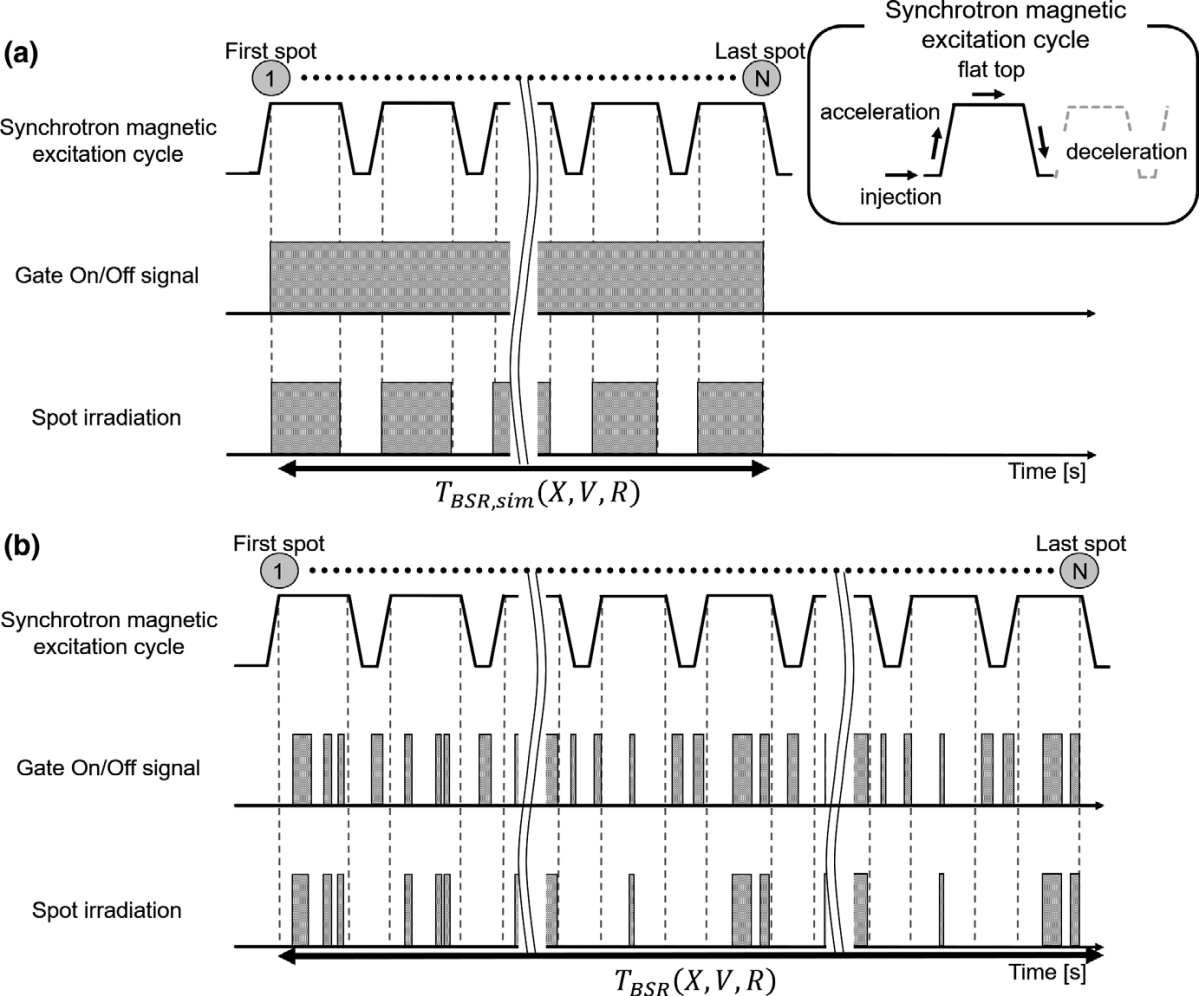
Schematic illustration that shows the timing of spot irradiation based on the difference between the synchrotron magnet excitation patterns for ideal environment (a) and actual treatment (b). N: number of spots in the treatment plan, $T_{BSR,sim}\left(X,V,R\right)$: simulated beam delivery time under the ideal synchrotron operating condition, $T_{\mathrm{BSR}}\left(X,V,R\right)$: actual beam delivery time from the proton beam delivery machine log data.

To compare the beam delivery time when the gate irradiation was not performed as part of the same treatment planning, we evaluated the difference between $T_{\mathrm{BSR}}\left(X,V,R\right)$ and the simulated beam delivery time under the ideal environment ($T_{BSR,sim}\left(X,V,R\right)$). Figure 5 illustrates the difference between the synchrotron magnetic excitation cycles for an ideal environment and actual treatment. Here, the ideal environment is a situation where the gate on/off signal is always output as shown in Fig. 5A. The value of $T_{\mathrm{BSR}}\left(X,V,R\right)$ was quantitatively analyzed using proton beam delivery machine log data, and $T_{BSR,sim}\left(X,V,R\right)$ was calculated from the in‐house simulation system, which used the treatment planning data. In the in‐house simulation system, it is possible to simulate the estimated $T_{BSR,sim}\left(X,V,R\right)$ using the synchrotron magnetic excitation cycles and the number of spots and layers. The details of the calculation parameters in the synchrotron magnetic excitation cycle, such as injection time, maximum flat top period, maximum spill length, acceleration and deceleration times, and scan speed, are described in a previous report.^9^

### 2.6 Evaluation of frequency corresponding to baseline shift or drift and beam delivery efficiency

To treat a moving tumor, it is important to understand the frequency corresponding to the baseline shift or drift and the beam delivery efficiency. We defined the frequency of intra‐field adjustment corresponding to the baseline shift or drift as the ratio of the total number of sessions to the number of sessions of intervention or adjustment to address baseline shift or drift, such as couch moving. In this study, we used the proton beam delivery machine log data to analyze the patient treatment process flow.

**Fig. 6:**
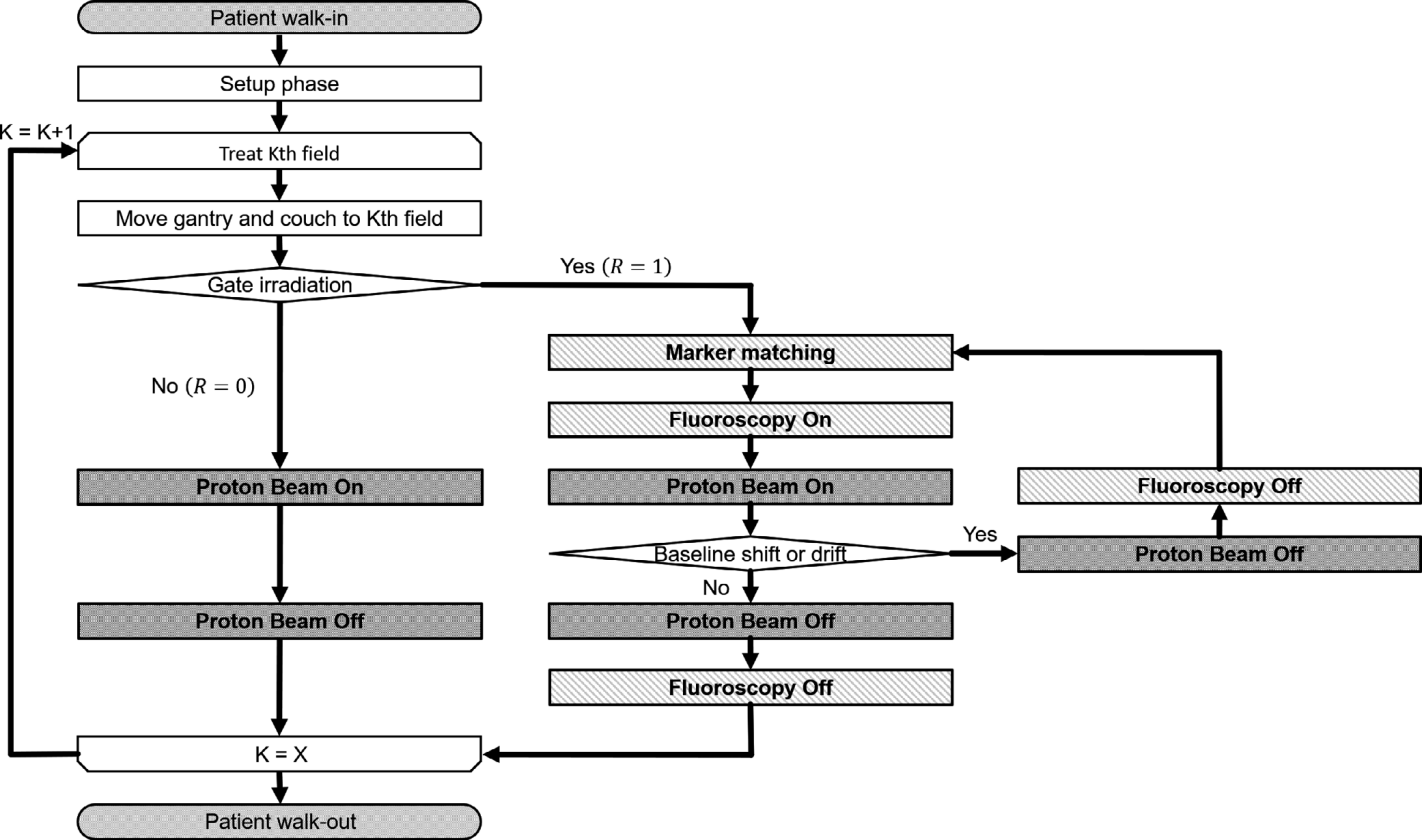
Flowchart of the RGPT machine log system in one treatment session with *X* treatment fields. *K* is the index number of the treatment field and *R* represents the usage of the gating function..

A flowchart of the RGPT machine log system in one treatment session with *X* treatment fields without the gating function (*R* = 0) and with the gating function (*R* = 1) is shown in Fig. 6. In the RGPT machine log system, if we identified baseline shift or drift during the beam delivery, a characteristic signal (redo bone matching) corresponding to the baseline shift or drift was recorded between the beam‐on signal for the first spot and the beam‐off signal after the beam delivery to the last spot. Thus, we analyzed the frequency of intra‐field adjustments corresponding to the baseline shift or drift for each category. We also defined the beam delivery efficiency of the RGPT system as the ratio of the total number of the gate on/off signals to the number of gates on signals during the beam delivery, as recorded in the log data. We evaluated the size of intra‐field adjustment as the amount of couch movement using the record of the Patient Positioning Image and Analysis System (PIAS) (Hitachi, Ltd., Tokyo, Japan).

## 3 RESULTS

### 3.1 Evaluation of beam delivery time

We analyzed 127 sets of treatment log data from the proton beam delivery machine. In total, 2810 sessions were delivered with gated irradiation. As listed in Table 2, the mean $T_{\mathrm{BSR}}\left(X,V,R\right)$ was 7.1 min, the mean $T_{BSR,sim}\left(X,V,R\right)$ was 2.9 min, and the difference between $T_{BSR,sim}\left(X,V,R\right)$ and $T_{\mathrm{BSR}}\left(X,V,R\right)$ was 4.3 min.

**Table 2:** Summary of the beam delivery time in ideal environment and actual treatment

Categories	Continuous [min]	Gated delivery [min]	Difference [min]
	Mean ± SD	Mean ± SD	Mean ± SD
Prostate	2.9 ± 0.4	5.3 ± 1.3	2.4 ± 1.1
Liver	2.8 ± 1.5	8.7 ± 5.6	5.9 ± 4.4
Pancreas	2.5 ± 0.4	13.2 ± 7.0	10.7 ± 6.7
Lung	3.7 ± 1.3	9.7 ± 3.1	6.0 ± 1.9
Adrenal gland	1.7 ± N.A.	5.6 ± N.A.	2.0 ± N.A.
Total	2.9 ± 1.0	7.1 ± 4.5	4.3 ± 4.0

### 3.2 Evaluation of frequency corresponding to baseline shift or drift and beam delivery efficiency

**Fig. 7:**
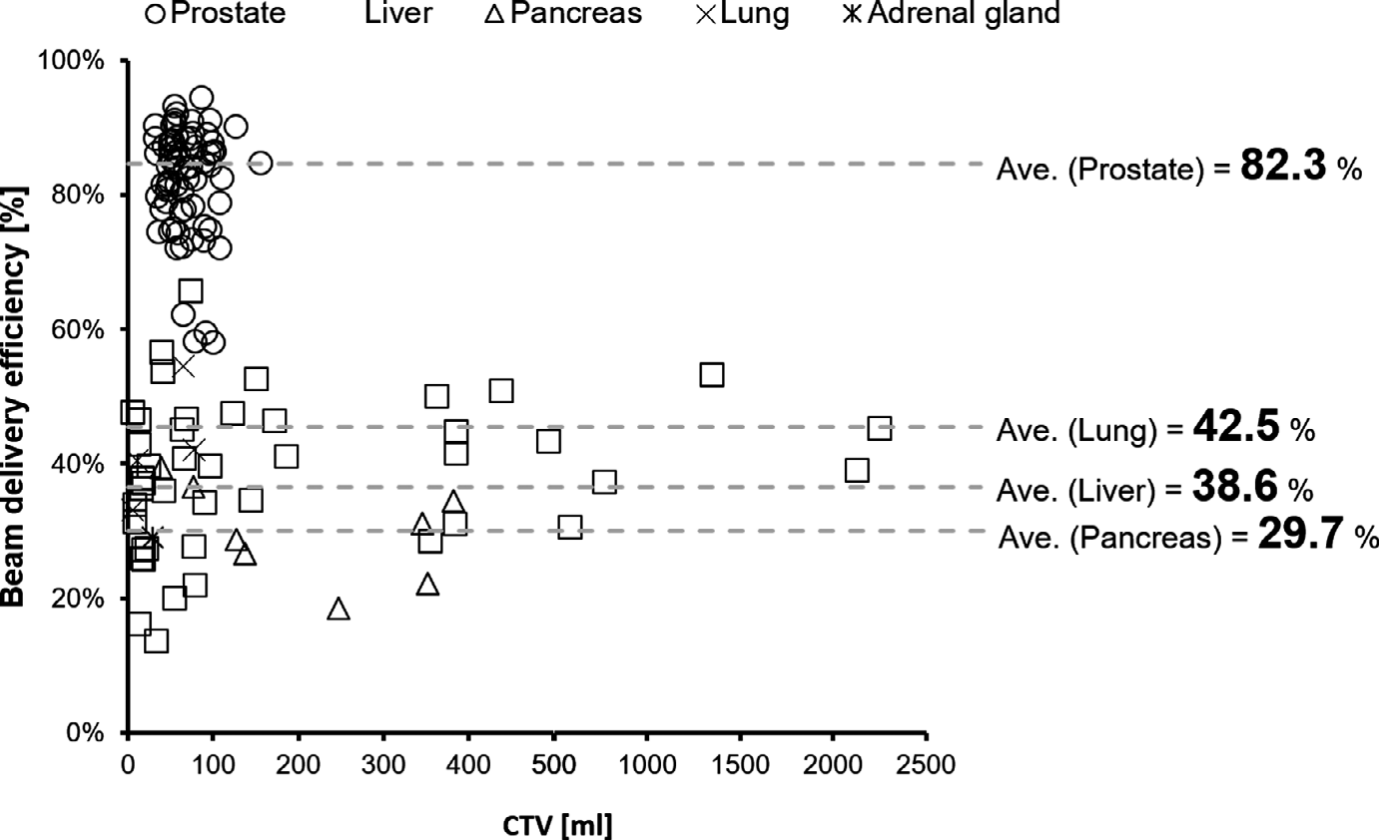
Scatter plot showing the relationship between the CTV and beam delivery efficiency.

During the treatment with gating (see Table 3) the average frequency of intra‐field adjustment corresponding to the baseline shift or drift was 21.7%, while the average beam delivery efficiency was 61.8%. Figure 7 shows the relationship between the CTV and beam delivery efficiency.

**Table 3:** Results of the frequency of intra‐field adjustment corresponding to the baseline shift or drift, the beam delivery efficiency, and the size of intra‐field adjustment

Categories	Frequency corresponding to the baseline shift or drift [%]	Beam delivery efficiency [%]	Size of intra‐field adjustment [cm]
	Mean ± SD	Mean ± SD	Mean ± SD
Prostate	18.5% ± 16.1%	82.3% ± 8.1%	0.28 ± 0.17
Liver	20.1% ± 21.0%	38.6% ± 11.0%	0.34 ± 0.29
Pancreas	49.3% ± 12.4%	29.7% ± 7.1%	0.34 ± 0.13
Lung	45.0% ± 19.1%	42.5% ± 8.9%	0.28 ± 0.09
Adrenal gland	20.0% ± N.A.	29.0% ± N.A.	0.14 ± 0.20
Total	21.7% ± 19.5%	61.8% ± 24.3%	0.30 ± 0.20

The average size of intra‐field adjustment corresponding to the baseline shift or drift was 0.30 cm. The average change in the position of the treatment couch during the beam delivery time was 0.03 cm.

## 4 DISCUSSION

Currently, real‐time image acquisition using biaxial fluoroscopy devices is clinically available not only from Hitachi’s RGPT technology but also through other vendors. For example, the treatment is provided to lung and liver cancer patients using markerless tumor tracking with a carbon‐ion pencil beam scanning system.^30^ The number of patients that can be treated each day in particle therapy facilities is determined by various factors, including the patient setup phase and the beam delivery phase.^18^, ^19^, ^20^ One way to increase the patient throughput is to decrease the beam delivery time, especially for gating irradiation. It is important to understand the processes underlying the gating irradiation. The findings of this study will be useful for particle beam therapy facilities where there is a need to treat more patients in a limited time, to predict the patient throughput.

Careful observation of the location of fiducial markers has shown that intra‐field adjustments of the patient couch with the RTRT system are useful for maintaining treatment accuracy within ±2.0 mm despite the baseline shift or drift.^28^ In the results for prostate cancer patients, it was observed that intra‐field adjustments during the 10‐min period after initial patient setup were required in over 10% of cases (AP direction: 14.2%, SI direction: 12.3%, and LR direction: 5.0%). Although this depends on the patient, similar to the previous report, the frequency corresponding to baseline shift or drift for prostate cancer, which accounted for 57% of the cases in this study, was 18.5% (Table 3).

Table 2 shows the results for actual beam delivery time and simulated beam delivery time with gating obtained under conditions close to those of an ideal environment. Moreover, the beam delivery efficiency was 61.8% in this study (Table 3). Tsunashima et al. investigated the efficiency of respiratory‐gated proton beam delivery and found that the beam delivery time for respiratory gating with a 30% duty cycle was 2–5 times longer than nongated proton beam delivery.^6^ In our results, the actual beam delivery time with gated irradiation was 2.5 times higher than the simulated data for the same treatment plan in an ideal environment. Our study shows that the synchrotron‐based RGPT system can realize a similar beam delivery time as respiratory‐gated proton beam delivery. Our results also appear to indicate that the beam delivery efficiency does not depend on the CTV (Fig. 6).

This study had some limitations. One limitation was the difference in proton pencil beam scanning methods. The beam scanning process in the lateral plane is typically performed in different ways.^31^, ^32^, ^33^, ^34^, ^35^ In contrast, this study on the synchrotron‐based RGPT system was focused on the spot‐scanning proton beam delivery with an inserted fiducial marker and gating. Thus, we could not confirm that the results would be the same for all beam delivery methods.

Another limitation was the beam delivery time, which may be too long in the era of proton therapy as an external beam radiation therapy. Because $T_{\mathrm{BSR}}\left(X,V,R\right)$ depends on the number of energy layers and spots,^18^, ^19^ a shorter $T_{\mathrm{BSR}}\left(X,V,R\right)$ reduces the treatment uncertainty and cost for moving tumors. The time to switch energy layers is approximately 2 s depending on the beam energy and makes up over 70% of $T_{\mathrm{BSR}}\left(X,V,R\right)$; thus, reducing the number of energy layers has a significant effect on $T_{\mathrm{BSR}}\left(X,V,R\right)$.^19^, ^36^

There are several methods for shortening $T_{\mathrm{BSR}}\left(X,V,R\right)$. One is to physically expand the proton pencil beam. When calculating a uniform dose distribution with a sharp proton pencil beam, an effective approach in order to shorten $T_{\mathrm{BSR}}\left(X,V,R\right)$ is to reduce the number of energy layers and spots by enlarging the proton pencil beam.^37^ A mini‐ridge filter (MRF) or ripple filter is useful for reducing the number of energy layers, which increase the peak width at low energy.^37^, ^38^, ^39^, ^40^, ^41^ For example, the MRF is used to reduce the ripple of the spread‐out Bragg peak (SOBP) in proton passive scattering beam delivery^39^ or is applied to layer stacking beam irradiation in carbon‐ion therapy.^40^ Matsuura et al developed and evaluated a short‐range applicator with an MRF for treating superficial moving tumors.^41^ Clinically, we have used this applicator with and without gated irradiation.

The structure of the short‐range applicator in the RGPT system limited the maximum irradiation field. The proton beam without the short‐range applicator has a maximum irradiation field size of 30 × 40 cm at the isocenter. In contrast, the proton beam with the short‐range applicator has a maximum irradiation field size of 14 × 14 cm at the isocenter because it must be situated a certain distance away so as not to block the X‐ray FOV. Therefore, it cannot be applied to all tumor sites. In this study, this short‐range applicator was used for gated irradiation in only 15 out of 74 treatment plans.

The other method for shortening $T_{\mathrm{BSR}}\left(X,V,R\right)$ is to reduce the number of layers and spots during the treatment plan. Van de Water et al. shortened $T_{\mathrm{BSR}}\left(X,V,R\right)$ by reducing the proton energy layers during treatment plan optimization.^42^ They shortened the beam delivery time by 16%–38% for oropharyngeal and prostate cases. Thus, the number of layers and spots can be reduced by optimization.

The final limitation was the method of operating the synchrotron accelerator to supply protons for the target. For our synchrotron, only one proton beam energy could be delivered per spill; this is called single energy extraction. Because of this, the synchrotron needed to discard all remaining protons and accelerate new protons for the next energy layer, even if only a few protons were delivered in an energy layer, which may not be efficient. Multi‐energy extraction can deliver multiple discrete energies within a single spill.^43^, ^44^, ^45^ As mentioned above, it takes approximately 2s to change the next spill,^36^ and there are many pauses during the beam delivery that depend on the gate signal, as shown in Fig. 5. There are no reports in the literature on combining the synchrotron‐based RGPT system and multi‐energy extraction. Thus, further research is required.

## 5 CONCLUSION

In this retrospective study, we quantitatively analyzed the proton beam delivery machine log data. Based on our clinical experience with a synchrotron‐based RGPT system, we determined the beam delivery time, the frequency of intra‐field adjustments corresponding to the baseline shift or drift, and the beam delivery efficiency. To maintain the treatment accuracy within ±2.0 mm, alterations corresponding to the baseline shift or drift were required in approximately 20% of cases, suggesting that real‐time monitoring and adjustments of the couch position are essential. Further improvements in beam delivery efficiency may be realized by shortening the beam delivery time.

## AUTHOR’S CONTRIBUTIONS

TY conceived this study. SS supervised the project and guarantor of the data. SS, TH, KN, NK, HT, KY, and HS treated and followed‐up the patients. TY, SS, MT, ST, MT, ST, YI, TM, HT, KH, and KU analyzed the data. TY wrote the first draft of the paper. All authors contributed to the drafting and editing of the manuscript and approved the final version.

## CONFLICTS OF INTEREST

Dr. Shirato reports grants from Hitachi, Ltd. and Shimadzu Corporation during the study and has licensed patents titled “Moving body pursuit irradiating device and positioning method using this device” (US6307914B1) and “Charged particle beam system” (US9757590). Dr. Umegaki reports grants from Hitachi, Ltd. during the study and has licensed patents titled “Charged particle beam system” (US9757590) and “Radiotherapy control apparatus and radiotherapy control” (US9616249). Dr. Shimizu reports grants from Hitachi, Ltd. during the study and has licensed patents titled “Charged particle beam system” (US9757590) and “Radiotherapy control apparatus and radiotherapy control program” (US9616249). The other authors have no relevant conflicts of interest to disclose.
